# Omega-3 fatty acids ameliorate doxorubicin-induced cardiorenal toxicity: *In-vivo* regulation of oxidative stress, apoptosis and renal Nox4, and *in-vitro* preservation of the cytotoxic efficacy

**DOI:** 10.1371/journal.pone.0242175

**Published:** 2020-11-12

**Authors:** Dalia Saleh, Marawan Abdelbaset, Azza Hassan, Ola Sharaf, Sawsan Mahmoud, Rehab Hegazy

**Affiliations:** 1 Department of Pharmacology, Medical Division, National Research Centre, Giza, Egypt; 2 Department of Pathology, Faculty of Veterinary Medicine, Cairo University, Giza, Egypt; National Institutes of Health, UNITED STATES

## Abstract

This study examines the protective effects of omega‐3 fatty acids (OMG), a frequently used nutritional therapy in cancer patients, against doxorubicin (DOX)‐induced acute cardiorenal toxicity in rats, and evaluates the cytotoxic activity of DOX when used with OMG against breast cancer cell line. Five groups of rats were treated for 4 consecutive weeks with vehicle (groups I & II), or OMG (25, 50 or 100 mg/kg/day, po; groups III, IV & V, respectively). After twenty-four hours, the last four groups were injected with DOX (200 mg/kg, ip). In DOX-treated rats, the altered ECG, serum cardiac and renal function biomarkers, and histopathological features indicated the induction of cardiorenal toxicity. Increased oxidative and apoptotic markers in both organs was observed, with elevated renal contents of NADPH-oxidase-4 (Nox4) and renin. OMG pretreatment improved those DOX-induced impairments in a dose-dependent manner, and showed antioxidant and antiapoptotic effects with regulation of renal Nox4 expression. The *in-vitro* study showed preservation of the cytotoxic activity of DOX on MCF7 cell line in the presence of OMG. The data suggests OMG for protection against acute DOX‐induced cardiorenal damage without affecting the latter antitumor activity. It proposes regulation of oxidative stress, Nox4 activity and apoptosis as contributing protective mechanisms.

## 1. Introduction

Doxorubicin (DOX) is an antineoplastic drug widely used in clinical practice to treat a wide range of malignancies [1]. However, the clinical use of DOX is restricted by its undesirable side effects, especially cardiotoxicity and nephrotoxicity [2,3]. Oxidative damage to cellular components is believed to be a major factor responsible for DOX-induced toxicity [1,4,5]. Induction of apoptosis is also among the mechanisms involved in DOX-induced toxic effects [6].

The exact mechanism by which DOX treatment differentially affects the cardiac and renal function is obscure [7]. Also, it is incomprehensible whether DOX-induced cardiaotoxicity precedes or follows nephrotoxicity. Cardiotoxicity usually appears in a significant dose-dependent manner in the form of congestive heart failure, and it develops within a short period after initiation of therapy in humans. It is characterized by a progressive degeneration of heart muscle [7]. DOX-induced renal toxicity, besides being a part of a multiorgan damage mediated mainly through reactive oxygen species (ROS) formation, it may be mediated directly through accumulation of DOX preferentially in the kidney. Additionally, DOX-induced cardiotoxicity may lead to modulation of blood supply to the kidney and alter the detoxification processes, respectively, thus indirectly contributing to DOX-induced nephropathy [8]. Remarkably, although the reported NADPH oxidase-4 (Nox4)-stimulating activity of DOX [9], and the emerging evidences support the role of Nox4 in different nephropathogenesis [10,11], few data correlates this Nox4-stimulating effect of DOX with its nephrotoxic activity.

Omega-3 fatty acids (OMG) are considered immunonutrients and are frequently used in the nutritional therapy of cancer patients due to their plentiful biological effects, and for alleviation of many cancer-related complications [12]. These fatty acids, including eicosapentaenoic acid (EPA) and docosahexaenoic acid (DHA), are incorporated in many parts of the body, including cell membranes, and play a role in cell signaling and the anti-inflammatory and anti-oxidant processes [13,14]. Moreover, they are precursors of more active metabolites that have many beneficial effects in preventing and/or treating several diseases [15], most importantly, the cardiovascular diseases [16–18]. Also, the renoprotective effect of OMG has been also established [19]. Clinical studies suggest that long-term treatment with OMG fatty acids improves renal function and lowers the risk of death or end-stage renal disease [20].

The present study investigated the potential protective effects of OMG against DOX-induced cardiorenal toxicity in rats. It evaluated some possible underlying mechanisms that may be involved in the DOX toxicity and OMG protective effects, including oxidative stress, apoptosis, and renal Nox4 pathways. The study also assessed the cytotoxic activity of DXO *in vitro* for any interaction that may hinder its anti-cancer activity when combined with OMG.

## 2. Materials and methods

### 2.1. Drugs and chemicals

Doxorubicin (DOX), Sulforhodamine-B (SRB) and dimethyl sulfoxide (DMSO) were purchased from Sigma-Aldrich (USA). Fish oil omega-3 fatty acids (OMG) were obtained from BIOVEA (Egypt); each 100 ml of this fish oil provides 95 mg of total OMG comprising 18 mg EPA, 12 mg DHA, and other fatty acids. Dulbecco's Modified Eagle Medium (DMEM medium), penicillin-streptomycin (×100), 0.25% trypsin-EDTA, phosphate buffered saline (PBS) and fetal bovine serum were purchased from Lonza Group Ltd. (Switzerland). All other chemicals were of the highest available analytical grade.

### 2.2. Animals

Adult female Wistar albino rats weighing 180–200 g were used in this study. They were obtained from the Animal House Colony of the National Research Center (NRC, Egypt). Animals were kept under standardized conditions with a 12-h light and dark cycle, and were allowed food and tap water ad libitum. They were treated according to the national and international ethics guidelines stated by the ethics committee of NRC. As well, this study has been approved by the ethics committee of NRC, and all procedures and experiments were performed according to a protocol approved by it.

### 2.3. Cell culture

Human breast cancer cell line MCF7 was obtained from VACSERA (Cairo, Egypt). Cells were maintained in DMEM media supplemented with 100 mg/mL of streptomycin, 100 units/mL of penicillin and 10% of heat-inactivated fetal bovine serum in humidified, 5% (v/v) CO2 atmosphere at 37°C.

### 2.4. Experimental design

Rats were allocated into five groups each consisting of 7–8 animals. Treatments were orally administered for 4 consecutive weeks as follows: Groups I and II received distilled water, while groups III, IV and V received OMG (25, 50 and 100 mg/kg/day, p.o.), respectively. Twenty-four hours after the last doses of OMG, each rat of groups II, III, IV and V was given a single intraperotoneal injection of DOX (200 mg/kg).

### 2.5. Electrocardiographic (ECG) measurements

Forty-eight hours after DOX injection, the body wieght for each of the animals was measured and the rats were anaesthetized with thiopental (5 mg/kg, i.p.) and kept warmed with a heating lamp to prevent the incidence of hypothermia. Subcutaneous peripheral limb electrodes were inserted for ECG recording (HPM 7100, Fukuda Denshi, Japan) for 1 min. Heart rate, P duration, QTc, and ST Height were monitored using ECG Powerlab module which consists of Powerlab/8sp and Animal Bio-Amplifier (Australia), in addition to Lab Chart 7 software with ECG analyzer [21,22].

### 2.6. Serum collection and tissue preparation

After ECG recording, blood samples were collected from the retro orbital sinus using heparinized capillary tubes under *ip* anesthesia with ketamine–xylazine (K, 100 mg/kg; X, 10 mg/kg) [23] for serum separation. The rats were then sacrificed by cervical dislocation under the same anethsethia, the heart and both kidneys of each animal were rapidly isolated, washed with ice-cold saline, dried and weighted. Then, weighed parts from all organs were homogenized in cold buffer, using a homogenizer (Heidolph Diax 900, Germany) to prepare 10% homogenate. The serum and homogenates were used for biochemical assays.

### 2.7. Serum and tissue biochemical analysis

Sera stored at –20°C were used for the estimation of serum cardiac and renal injury boimarkers such as creatine kinase-myocardial band (CK-MB or CK for short) [24], urea [25], and creatinine [26]. A highly sensitive quantification of CRP (hsCRP) in serum was also performed using a specific rat enzyme-linked immunosorbent assay (ELISA) kit (Biovendor, Czech Republic) as per the manufacturer’s instructions.

Cardiac and renal tissues homogenate were used for determination of lipid peroxidation measured as malondialdehyde (MDA) besides reduced glutathione (GSH), as oxidative stress markers, using specific diagnostic kits (Biodiagnostic, Egypt). Cardiac concentrations of atrial natriuretic peptide (ANP) and interleukin-6 (IL-6), as well as renal contents of reduced nicotinamide adenine dinucleotide phosphate (NADPH) oxidase-4 (Nox4) and renin were measured using the specific rat ELISA kits (Biovendor, Czech Republic) according to the manufacturer’s instructions. The data were normalized to tissue weight by multiplying by a dilution factor used in the preparation of the tissue homogenates.

### 2.8. Histopathological examination

Tissue specimens from the heart and kidneys of control and treated rats were fixed in 10% neutral buffered formalin and routinely processed. Paraffin embedded tissues were cut into 4μm thick sections and stained with hematoxylin and eosin (H&E) for routine histopathological examination. A total of ten fields per group were blindly examined. For assessment of cardiotoxicity, the cardiac tissue sections were stained with Toluidine blue for demonstration of cardiomyocyte vacuolation characteristic of doxorubicin-induced cardiomyocyte injury [27]. Cardiomyocyte vacuolation was semiquantitvely graded on a score scale ranged from 0 to 3, based on the percentage of cardiomyocytes displaying vacuolization of sarcoplasm, in which score 0 denote no vacuolation; score 1 denote minimal grade (< 5%); score 2 denote moderate grade (5–30%) and score 3 denote severe grade (˃35%).

For assessment of nephrotoxicity, a scoring scale graded from 0 to 4 was used according to the method described by Herman et al, 1988, in which 0 = the tissue appeared normal, 1 = minimal lesion, 2 = mild lesion, 3 = moderate lesion and 4 = severe lesion. The histopathological criteria used in evaluation of renal lesions were tubular degeneration and /or necrosis, tubular dilatation, deposition of renal cast in the tubular lumina and interstitial inflammatory cell infiltrates.

### 2.9. Immunohistochemical investigation

For Caspase 3 and tumor protein P53 immune staining, the paraffin-embedded sections were dewaxed, rehydrated and incubated in 3% hydrogen peroxide for inhibition of endogenous peroxidase. The sections were then incubated with rabbit anti-cleaved caspase 3 (Abcam, 1:100 Cambridge, MA, USA) and mouse monoclonal anti-P53 (Ventana) as the primary antibodies. Staining was visualized with DAB. Cells displaying brown cytoplasm and/or nuclei were considered positive. The immunohistochemical staining was graded on three point scoring system ranging from 0 to 2, depending on the distribution of area involved, in which score 0 represent ˃30% of area involvement, score 1 represent 30% - 60% and score 2 represent <60% [28].

### 2.10. Cytotoxicity assay

To study the effect of OMG on the cytotoxic profile of DOX, the dose response curve of DOX alone was assessed relative to its combination with omega 3 in MCF7 line. Cell viability was assessed by sulforhodamine B (SRB) assay in three replicates. Aliquots of 100 μL cell suspension were seeded at 2×103–5×103 cells per well in 96-well plates and incubated in complete media for 24 h. Cells were treated with another aliquot of 100 μL media containing DOX, OMG and their equimolar combinations at various concentrations (0.01, 0.1,1, 10 & 100 μM). After 72 h of drugs exposure, cells were fixed by replacing media with 150 μL of 10% TCA and incubation at 4°C for 1 h. The TCA solution was removed, and the cells were washed 5 times with distilled water. Aliquots of 70 μL SRB solution (0.4% w/v) were added and incubated in a dark place at room temperature for 10 min. Plates were washed 3 times with 1% acetic acid and allowed to air-dry overnight. Then, 150 μL of Tris–HCl (10 mM) was added to dissolve protein-bound SRB stain; the absorbance was measured at 540 nm using a microplate reader.

### 2.11. Statistical analysis

Data is presented as mean + S.E. Statistical analysis of the data was carried out using one way analysis of variance (ANOVA) followed by Tukey's multiple comparisons test to judge the difference between the various groups. Statistical significance was acceptable to a level of *p* < 0.05. Data analysis was accomplished using the software program GraphPad Prism, version 5 (GraphPad Software, USA).

Statistical analysis of IC50 values were calculated from concentration-response curves by Sigma Plot software, version 12.0 (System Software, USA), using an E-max model equation:

$$\%Cell\;viability=\left(100‐R\right)\;x\;\left(1‐\left({\left(D\right)}^{m}/K^{d}{}_{m}+{\left(D\right)}^{m}\right)\right)$$

Where (R) is the residual unaffected fraction (the resistance fraction), (D) is the drug concentration used, (K^d^) is the drug concentration that produces a 50% reduction of the maximum inhibition rate and (m) is a Hill-type coefficient. IC50 was defined as the drug concentration required to reduce absorbance to 50% of that of the control (i.e., K^d^ = IC50 when R = 0 and Emax = 100−R).

## 3. Results

### Effect of OMG on heart and kidneys relative weights

DOX did not alter the body weight (BW), heart (HW) and kidneys weight (KW) significantly and, subsequently, did not alter their relative weights. Oral treatment of DOX-injected rats with OMG also did not show any alteration in the tissues’ weights (Table 1).

**Table 1:** Effect of OMG on body weight, heart weight, kidney weight and their relative weight in DOX-treated rats.

Parameters Groups	BW (g)	HW (g)	KW (g)	RHW	RKW
**Normal**	119.2±5.93	0.488±0.032	1.072±0.05	0.413±0.02	0.00906±0.0003
**DOX**	107.0±3.97	0.626±0.020	1.078±0.12	0.589±0.04	0.009913±0.0011
**DOX+OMG (25mg/kg)**	101.4±11.60	0.394±0.074^@^	0.916±0.05	0.417±0.09	0.009532±0.0009
**DOX+OMG (50mg/kg)**	122.0±5.06	0.508±0.021	0.85±0.01	0.417±0.02	0.007061±0.0012
**DOX+OMG (100 mg/kg)**	123.8±3.46	0.550±0.017	1.09±0.05	0.453±0.02	0.009006±0.0006

Cardiorenal toxicity were induced in rats by a single intraperioneal injection of doxorubicin (DOX; 200 mg/kg, i.p) after 28 consecutive days of oral administration of omega 3 (OMG; 25, 50 and 100 mg/kg) fatty acids. Forty-eight hours after DOX injection, rats were weighed (BW) and their hearts (HW) and renal (RW) were also weighed after scarification. The relative heart (RHW) and kidney (KHW) weights to BW were calculated. Results are the mean ± SEM. Statistical analyses were carried out using ANOVA followed by Tukey's multiple comparisons test.

@, *P*< 0.05 compared with the DOX treated rat.

### 3.2. Effect of OMG on electrocardiographic measurements

**Fig 1:**
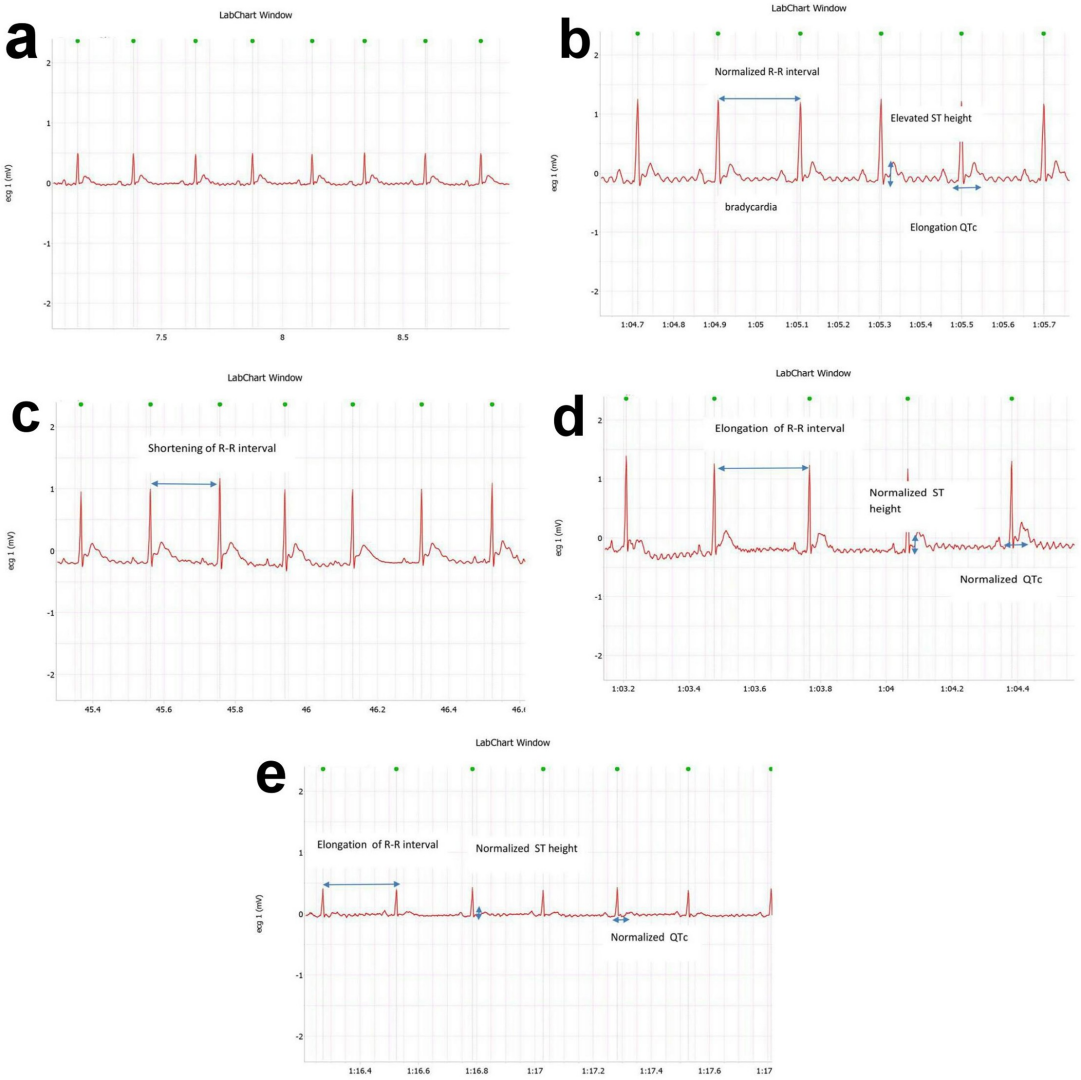
Effect of OMG fatty acids on ECG patterns in DOX-treated rats. Cardiorenal toxicity were induced in rats by a single intraperioneal injection of doxorubicin (DOX; 200 mg/kg,i.p) after 28 consecutive days of oral administration of omega 3 (OMG; 25, 50 and 100 mg/kg) fatty acids. Forty-eight hours after DOX injection, rats were anesthetized and the electrocardiography (ECG) was recorded for 1 min. (a) showing part of ECG of normal control group (b) showing part of ECG of DOX group with marked bradycardia, elongation of R-R interval, elongation of QTc interval, elevated ST height and depressed T amplitude as compared to normal control group (c) showing part of ECG of DOX+OMG 25 mg/kg group with normalized R-R interval, ST height and elongation of QTc interval (d) showing part of ECG of DOX+OMG 50 mg/kg group with elongated R-R interval and ameliorated of both QTc interval and ST height (e) showing part of ECG of DOX+OMG 100 mg/kg group with elongated R-R interval and bettered of both QTc interval and ST height.

The intra-peritoneal injection of DOX induced bradycardia with elongation of R-R interval, accompanied by elongation of corrected QTc, elevation of ST height and shortening of T amplitude as compared with normal group. However, treatment of DOX-injected rats with OMG (25, 50 or 100 mg/kg; p.o.) showed a marked dose-dependent ameliorative effect on QTc, ST height and T amplitude as compared to the positive control group (Fig 1 and Table 2).

**Table 2:** Effect of OMG on R-R interval, heart rate, QRS interval, QTc duration, P duration, ST height and T amplitude of DOX-treated rats.

Parameters Groups	ECG Parameters					
	R-R	Heart Rate (bpm)	QTc duration (s)	P amplitude (mV)	ST Height (mV)	T amplitude (mV)
**Normal**	0.18±0.008	312.8±9.56	0.11±0.0065	0.056±0.004	0.059±0.007	0.25±0.009
**DOX**	0.23±0.002*	253.4±3.10*	0.19±0.0071*	0.065±0.010	0.123±0.022*	0.07±0.019*
**DOX+OMG 25 mg/kg**	0.17±0.006^@^	352.7±11.87*^@^	0.18±0.010*	0.051±0.010	0.064±0.013^@^	0.16±0.0276
**DOX+OMG 50 mg/kg**	0.25±0.008*	245.2±8.36*	0.13±0.009^@^	0.053±0.037	0.0734±0.012^@^	0.19±0.044
**DOX+OMG 100 mg/kg**	0.25±0.008*	242.8±8.97*	0.14±0.014^@^	0.059±0.014	0.029±0.009^@^	0.17±0.048

Cardiorenal toxicity were induced in rats by a single intraperioneal injection of doxorubicin (DOX; 200 mg/kg,i.p) after 28 consecutive days of oral administration of omega 3 (OMG; 25, 50 and 100 mg/kg) fatty acids. Forty-eight hours after DOX injection, the body mass for each of the animals was measured and the rats were anaesthetized with thiopental and ECG recording for 1 minute. Heart rate, P duration, QTc, and ST Height were monitored.

Results are the mean ± SEM. Statistical analyses were carried out using ANOVA followed by Tukey's multiple comparisons test.

*, P< 0.05 compared with the normal control

@, P< 0.05 compared with the DOX treated rats.

### 3.3. Effect of OMG on biochemical assessments

Induction of cardiac toxicity with DOX has been noticed by the marked elevation in the serum CK level as compared to the normal control group by about 2.4 folds. Oral administration of OMG (25, 50 or 100 mg/kg) for 4 consecutive weeks to DOX-injected rats showed a suppression of the CK level by 25%, 27% and 57%, respectively, in a dose dependent manner. However, renal toxicity has been observed after DOX intraperitoneal injection by significance incrimination of serum levels of urea and creatinine by 2 and 2.6 folds respectively, as compared to normal control group. Administration of OMG (25, 50 and 100 mg/kg) for 4 consecutive weeks succeed to alleviate the serum levels of urea and creatinine by about 24%, 40% and 44%, respectively and 36%, 46% and 59%, respectively (Table 3).

**Table 3:** Effect of OMG on serum cardiac and renal function parameters in DOX-treated rats.

Parameters Groups	Serum CK-MB (U/l)	Serum Urea (g/dl)	Serum Cr (mg/dl)
**Normal**	28.11±2.85	13.11±1.03	17.33±1.78
**DOX**	67.13±0.54*	25.66±1.09*	62.66±5.26*
**DOX+OMG (25mg/kg)**	50.30±4.13*^@^	19.55±1.1*^@^	40.33±3.75*^@^
**DOX+OMG (50mg/kg)**	48.92±4.39*^@^	15.32±1.26^@^	34.08±3.12*^@^
**DOX+OMG (100 mg/kg)**	35.58±2.65*^@^	14.45±0.97^@^	25.84±2.18^@^

Cardiorenal toxicity were induced in rats by a single intraperioneal injection of doxorubicin (DOX; 200 mg/kg,i.p) after 28 consecutive days of oral administration of omega 3 (OMG; 25, 50 and 100 mg/kg) fatty acids. Forty-eight hours after DOX injection, blood samples were collected and sera were separated for determination of creatine kinase-myocardial band (CK-MB), urea, and creatinine (Cr). Results are the mean ± SEM. Statistical analyses were carried out using ANOVA followed by Tukey's multiple comparisons.

test.

*, P< 0.05 compared with the normal control

@, P< 0.05 compared with the DOX treated rats.

An elevation in level of lipid peroxidation evidenced by MDA was noticed in both cardiac and renal tissues in DOX-treated rats by about 2 and 1.73 folds, respectively. On the other hand, the cardiac and renal levels of GSH was reduced in DOX-treated rats by about 37% and 53%, respectively. Rats treated with OMG (25, 50 and 100 mg/kg) daily for 28 consecutive days showed a reduction in the cardiac MDA level by about 26%, 45% and 40%, respectively and a reduction in the renal MDA level by about 19%, 21% and 39%, respectively as compared to DOX-treated group. Moreover, rats treated with OMG (25, 50 and 100 mg/kg) exhibited an elevation in the cardiac and renal GSH levels by about 29%, 37% and 45%, respectively and by about 31%, 56% and 76%, respectively relative to DOX-treated group (Table 4).

**Table 4:** Effect of OMG on cardiac and renal oxidative stress biomarkers in on DOX-treated rats.

Parameters Groups	Cardiac MDA (nmol/g tissue)	Renal MDA (nmol/g tissue)	Cardiac GSH (mg/g tissue)	Renal GSH (mg/g tissue)
**Normal**	26.36±1.02	33.97±0.87	61.11±1.54	18.03±0.55
**DOX**	54.96±1.97*	58.97±0.56*	38.56±0.89*	8.58±0.18*
**DOX+ OMG (25mg/kg)**	40.86±1.65*^@^	47.59±0.16*^@^	49.93±1.64*^@^	11.24±0.19*^@^
**DOX+ OMG (50mg/kg)**	30.12±0.95^@^	46.09±2.22*^@^	52.68±1.71*^@^	13.38±0.45*^@^
**DOX+ OMG (100 mg/kg)**	33.41±2.54^@^	35.89±2.67^@^	56.05±1.83^@^	15.11±0.47*^@^

Cardiorenal toxicity were induced in rats by a single intraperioneal injection of doxorubicin (DOX; 200 mg/kg,i.p) after 28 consecutive days of oral administration of omega 3 (OMG; 25, 50 and 100 mg/kg) fatty acids. Forty-eight hours after DOX injection, the rats were scarified and the heart and kidneys were isolated, homogenized, and the malondialdhyde (MDA) and reduced glutathione (GSH) contents were measured in the homogenate. Results are the mean ± SEM. Statistical analyses were carried out using ANOVA followed by Tukey's multiple comparisons test.

*, P< 0.05 compared with the normal control

@, P< 0.05 compared with the DOX treated rats.

**Fig 2:**
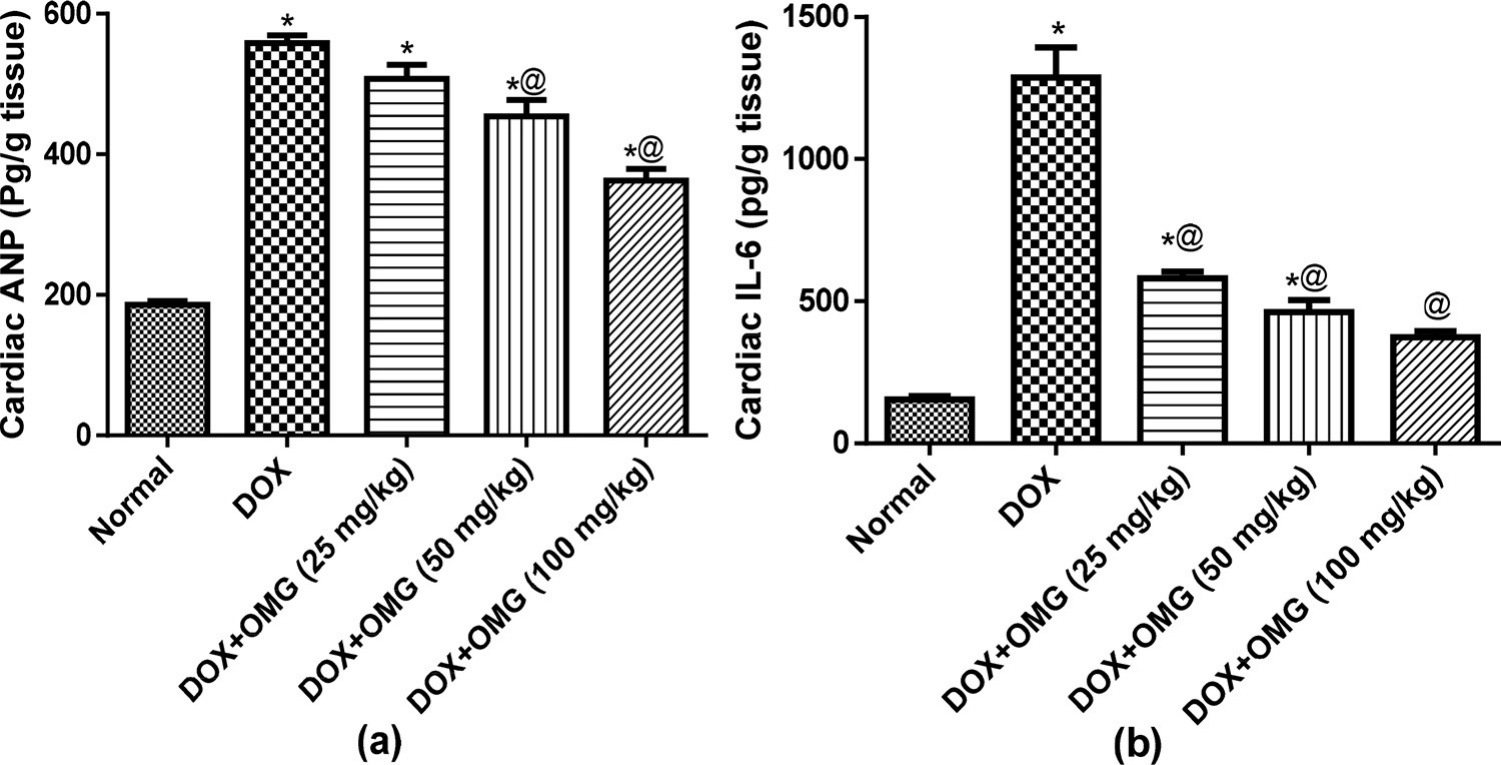
Effect of OMG on cardiac IL-6 (a) and ANP (b) in DOX-treated rats. Cardiorenal toxicity were induced in rats by a single intraperioneal injection of doxorubicin (DOX; 200 mg/kg,i.p) after 28 consecutive days of oral administration of omega 3 (OMG; 25, 50 and 100 mg/kg) fatty acids. Forty-eight hours after DOX injection, the rats were scarified and the heart weas isolated, homogenized, and the levels of **(a)** atrial natriuretic peptide (ANP) and **(b)** interleukin-6 (IL-6) were measured in the homogenate. Results are the mean ± SEM. Statistical analyses were carried out using ANOVA followed by Tukey's multiple comparisons test. *, P< 0.05 compared with the normal control; @, P< 0.05 compared with the DOX treated rats.

**Fig 3:**
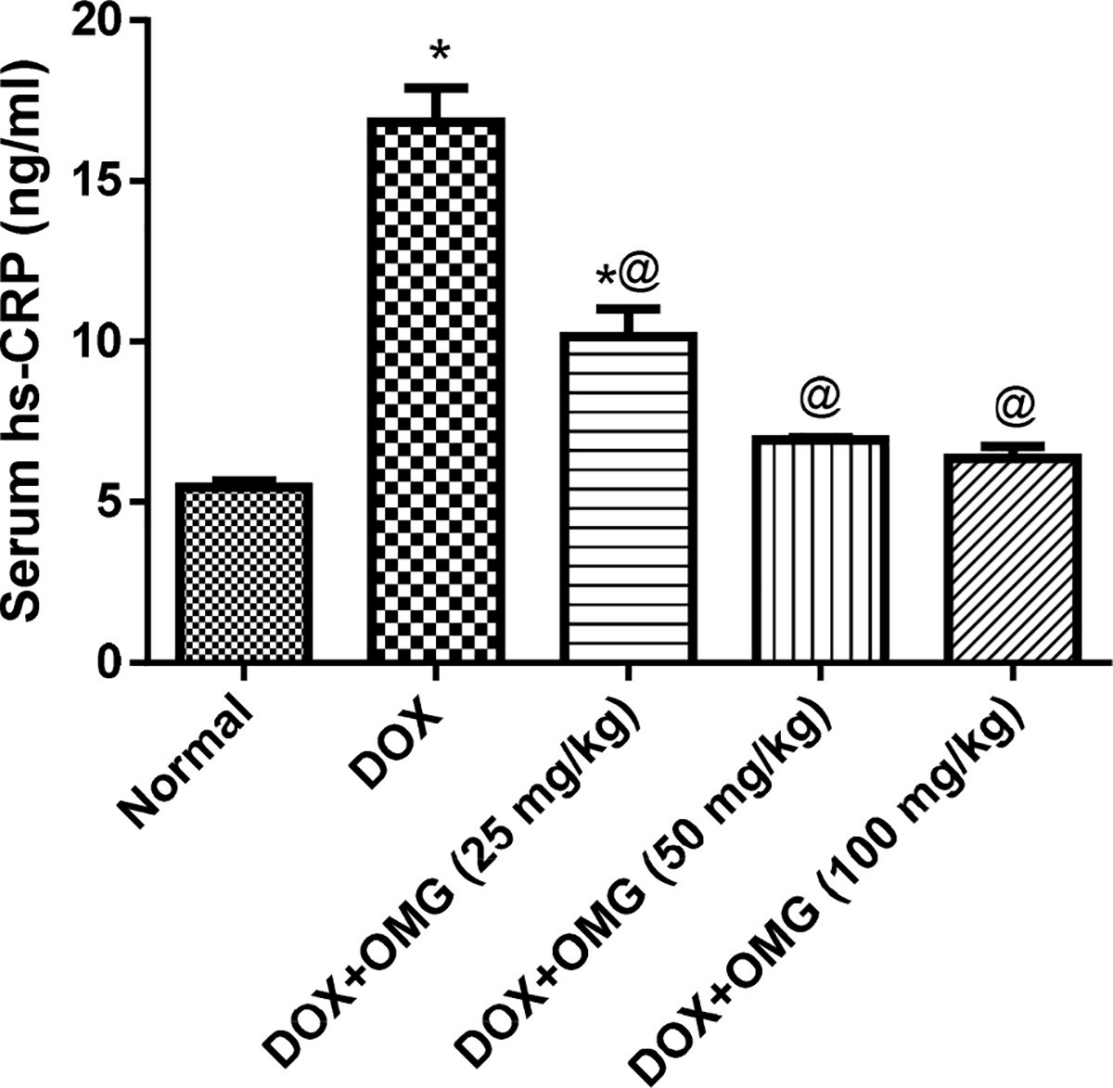
Effect of OMG on serum hs-CRP in DOX-treated rats. Cardiorenal toxicity were induced in rats by a single intraperioneal injection of doxorubicin (DOX; 200 mg/kg,i.p) after 28 consecutive days of oral administration of omega 3 (OMG; 25, 50 and 100 mg/kg) fatty acids. Forty-eight hours after DOX injection, blood samples were collected and sera were separated for determination of highly sensitive quantification of CRP (hs-CRP). Results are the mean ± SEM. Statistical analyses were carried out using ANOVA followed by Tukey's multiple comparisons test. *, P< 0.05 compared with the normal control; @, P< 0.05 compared with the DOX treated rats.

Doxorubicin injection was accompanied by a marked elevation of cardiac ANP levels by 3 folds as well as elevation of cardiac IL-6 levels by about 8 folds as compared to normal control group. Oral administration of OMG in its three dose levels showed a suppression of the cardiac IL-6 level by about 55%, 64% and 71%, respectively. Similarly, OMG (50 and 100 mg/kg) decreased the cardiac ANP level by about 17% and 35% (Fig 2). Similarly, the serum level of hs-CRP has been elevated by about 3 folds in DOX-treated rats while the rats treated with OMG (25, 50 and 100 mg/kg) for 28 consecutive days showed a suppression of the serum level of hs-CRP by about 40%, 59% and 62%, respectively as compared to the DOX-treated group (Fig 3).

**Fig 4:**
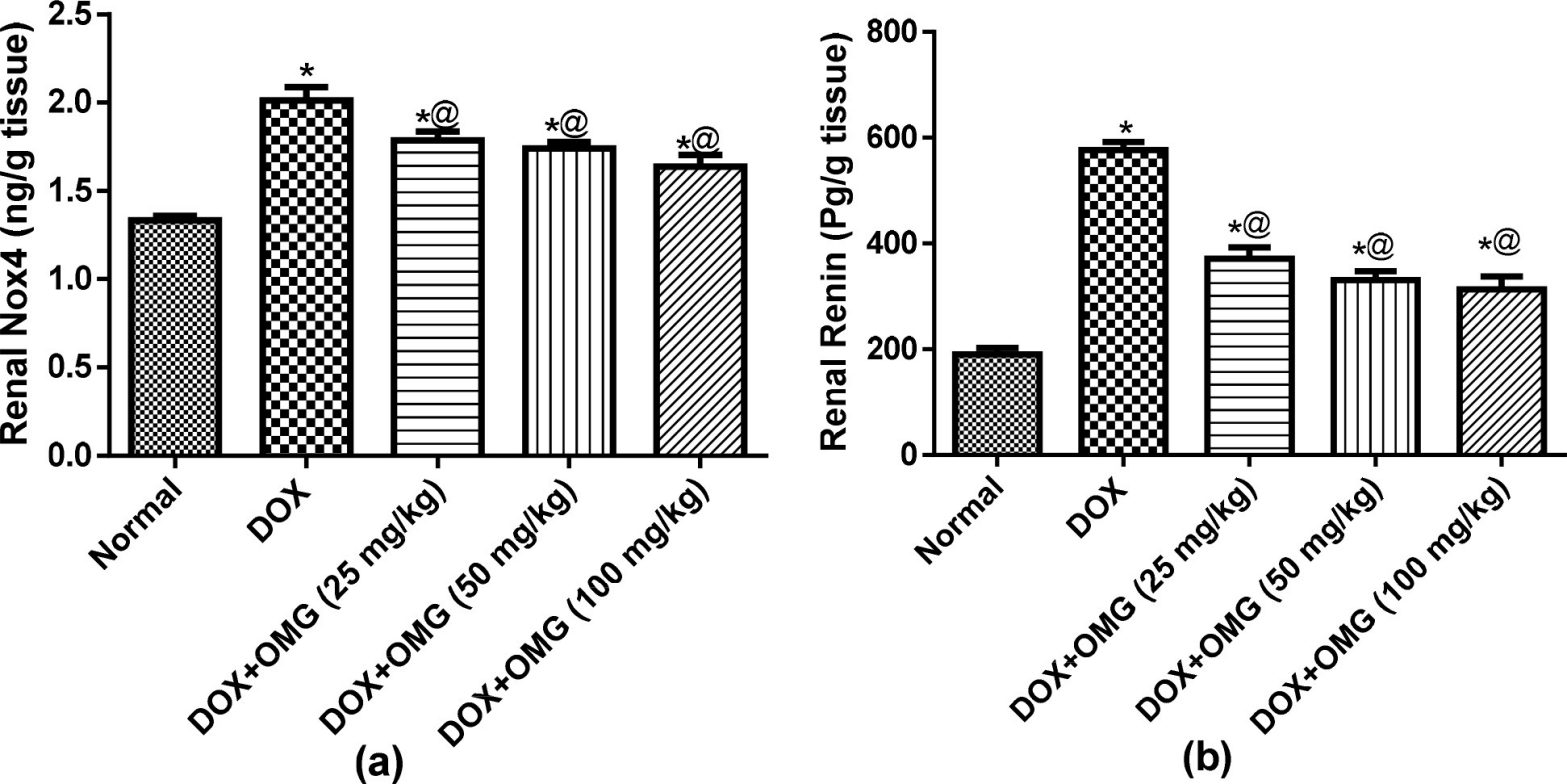
Effect of OMG on renal Nox4 (a) and renin (b) in DOX-treated rats. Cardiorenal toxicity were induced in rats by a single intraperioneal injection of doxorubicin (DOX; 200 mg/kg,i.p) after 28 consecutive days of oral administration of omega 3 (OMG; 25, 50 and 100 mg/kg) fatty acids. Forty-eight hours after DOX injection, the rats were scarified and the kidneys were isolated, homogenized, and the levels of **(a)** NADPH oxidase 4 (Nox4) and **(b)** renin were measured in the homogenate. Results are the mean ± SEM. Statistical analyses were carried out using ANOVA followed by Tukey's multiple comparisons test. *, P< 0.05 compared with the normal control; @, P< 0.05 compared with the DOX treated rats.

Induction of renal toxicity by DOX was associated with a significant elevation in renal Nox4 levels by about 50% as compared to the normal control group. However, this elevation has been ameliorated in a dose dependent manner in the groups treated daily with OMG (25, 50 and 100 mg/kg) for 28 consecutive days by about 11%, 13% and 18%, respectively (Fig 4A). On the other hand, DOX-treated group showed a prominent elevation in renal renin levels by 3 folds while treatment with OMG (25, 50 and 100 mg/kg) for 28 consecutive days declined renal renin levels by 36%, 43% and 46%, respectively in a dose dependent manner (Fig 4B).

### 3.4. Effect of OMG on histopathological examinations

The mean pathologic score recorded in the heart and kidneys of control and treated rats are illustrated in Table 5.

**Table 5:** Effect of OMG on cardiac and renal pathologic score of DOX-treated rats.

Parameters Groups	Cardiac Pathologic score	Renal Pathologic score
**Normal**	0.20±0.13	0.30±0.15
**DOX**	2.60*±0.16	3.20* ±0.24
**DOX+OMG (25mg/kg)**	2.20*^@^±0.20	2.30*^@^±0.42
**DOX+OMG (50mg/kg)**	1.70^@^±0.21	1.80^@^±0.32
**DOX+OMG (100mg/kg)**	1.40^@^±0.16	1.30^@^±0.21

Cardiorenal toxicity were induced in rats by a single intraperioneal injection of doxorubicin (DOX; 200 mg/kg,i.p) after 28 consecutive days of oral administration of omega 3 (OMG; 25, 50 and 100 mg/kg) fatty acids. Forty-eight hours after DOX injection, the rats were scarified and the heart and renal were isolated. Tissue specimens from the kidneys and heart of control and treated rats were fixed in 10% neutral buffered formalin and routinely processed. Paraffin embedded tissues were cut into 4μm thick sections and stained with H&E for routine histopathological examination. A total of ten fields per group were examined. For assessment of nephrotoxicity, a scoring scale graded from 0 to 4. Results are the mean ± SEM. Statistical analyses were carried out using ANOVA followed by Tukey's multiple comparisons test.

*, P< 0.05 compared with the normal control

^@^, P< 0.05 compared with the DOX treated rats.

Kidneys of normal control rats revealed normal histological structure of renal parenchyma with normal morphology of renal glomeruli and tubules (Fig 4A). In contrast, kidneys of DOX- treated rats revealed severe histopathological alterations particularly in the renal cortex. Glomeruli revealed glomerular congestion associated with presence of eosinophilic floculent material in Bowmanʼs space (Fig 4B). Renal tubules particularly proximal convoluted one revealed tubular nephrosis with extensive necrosis of their epithelial lining (Fig 4C) and intra luminal aggregation of cellular and protein cast (Fig 4D). Mild intertubular leukocytic cell infiltration (Fig 4E) and tubular dilatation were also demonstrated. Treatment with OMG revealed improvement at all doses level with most the significant improvement recorded in OMG (100mg/kg) +DOX group. Treatment with OMG (25mg/kg) +DOX revealed mild vacuolar degeneration of the surrounding renal tubules with intra luminal aggregation of renal cast (Fig 4F). Remarkable amelioration of the histopathological lesions was demonstrated in OMG (50mg/kg) +DOX and OMG (100mg/kg) +DOX, respectively, in which the renal tubular degeneration was confined to individual cells (Fig 4G and 4H, respectively).

**Fig 5:**
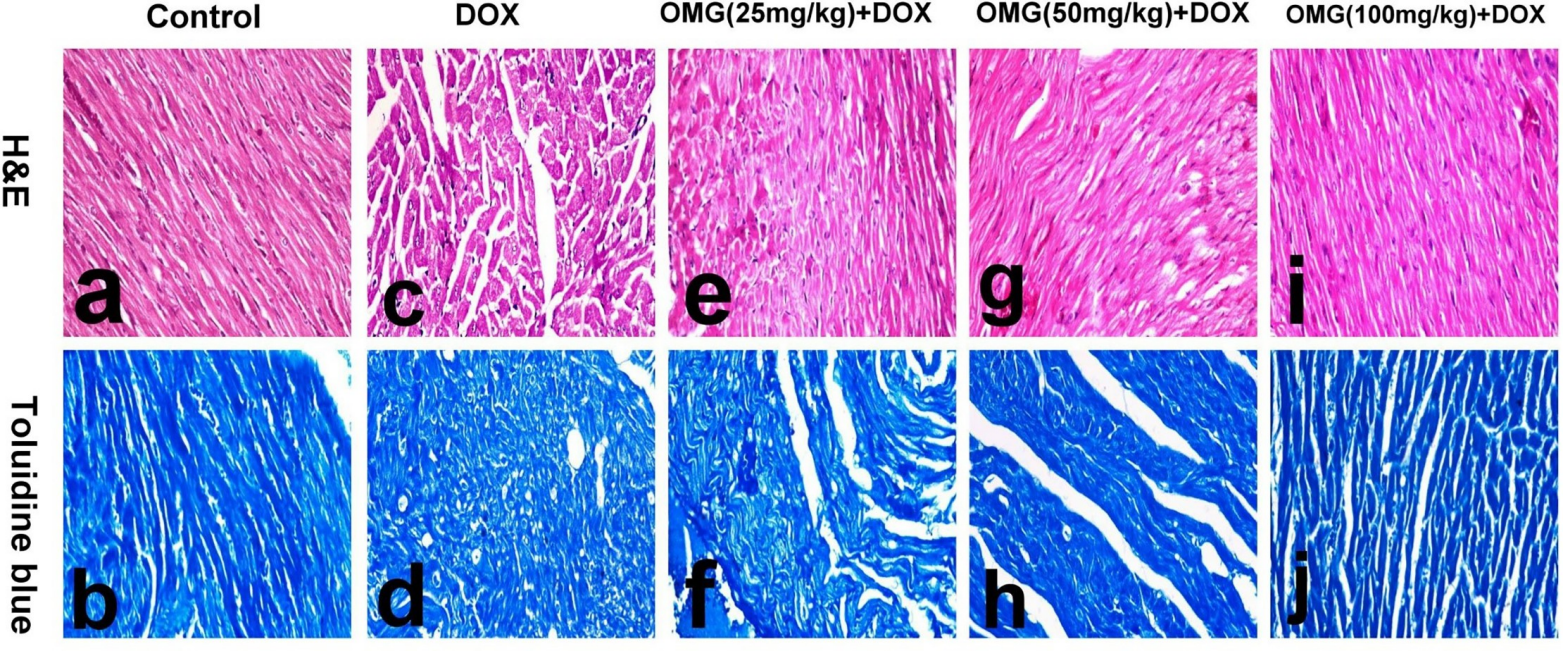
The findings of histopathological analysis of cardiac tissues of the rats. (a,b) normal rats showing normal cardiomyocyte (a) with no e of sarcoplasmic vacuolation (b), (c,d) DOX- treated rats showing vacuolization of cardiomyocyte, (e,f) OMG (25mg/kg)+DOX showing individual necrosis of cardiomyocyte with intensely eosinophilic cytoplasm (e) and cytoplasmic vacuolization (f), (g,h) OMG (50mg/kg)+DOX showing mild and focal vacuolation of cardiomyocyte and (i,j) OMG (100mg/kg)+DOX showing normal cardiomyocyte (i) with vacuolation of individual cardiomyocytes (j).(H&E for a, c, e, g, and i and Toluidine blue for b, d, f, h and j. 40X). DOX, doxorubicin, OMG, omega 3 fatty acids**.**

**Fig 6:**
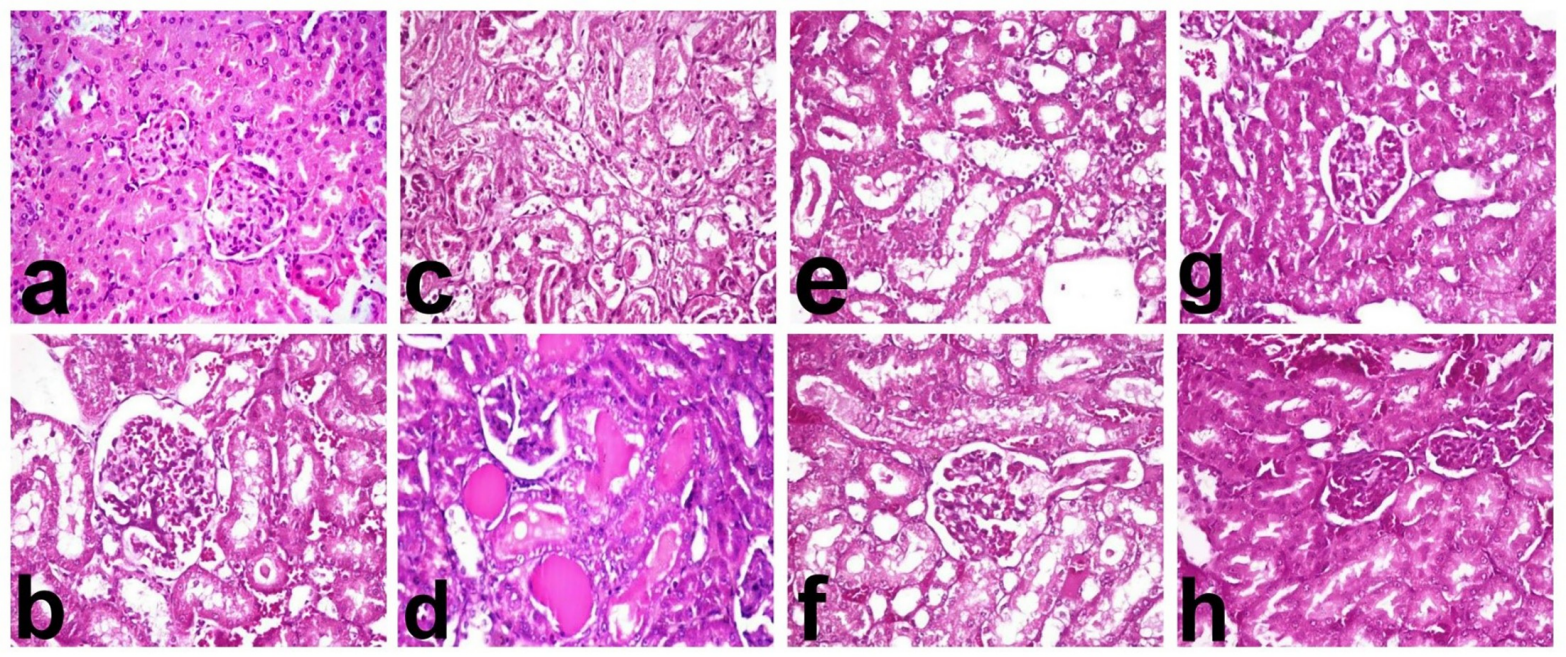
The findings of histopathological analysis of renal tissues of the rats. Kidneys of (a) normal rats showing normal renal glomeruli and tubules, (b, c, d) DOX- treated rats showing glomerular congestion (b), extensive necrosis of their epithelial lining (c), intra luminal aggregation of cellular and protein cast (d), and mild intertubular leukocytic cell infiltration (e), (f) OMG (25mg/kg)+DOX showing mild vacuolar degeneration of the surrounding renal tubules with intra luminal aggregation of renal cast, (g) OMG (50mg/kg)+DOX showing vacuolar degeneration of individual cells and (h) OMG (100mg/kg)+DOX showing normal renal tubules.(H&E, 40X). DOX, doxorubicin, OMG, omega 3 fatty acids.

Heart of control rats revealed normal cardiomyocyte with compact symmetrically arranged myofibrils and no evidence of sarcoplasmic vacuolization (Figs 5A and 6A). While, heart of DOX-treated rats revealed expanded vacuolization of cardiomyocyte sarcoplasm associated with loss of striations and individual necrosis of cardiomyocyte (Figs 5B and 6B). Mild regression of histopathological lesions was demonstrated in OMG (25mg/kg) +DOX 0.25 group with individual necrosis of cardiomyocyte and cytoplasmic vacuolization (Figs 5C and 6C). On the other side, marked attenuation of histapathological lesions was recorded in OMG (50mg/kg) +DOX group with mild and focal vacuolization of cardiomyocyte (Figs 5D and 6D). The cardiomyocyte restored its normal structure and appeared as normal as the control in OMG (100mg/kg) + DOX group (Figs 5E and 6E).

### 3.5. Effect of OMG on immunohistochemical analysis

**Fig 7:**
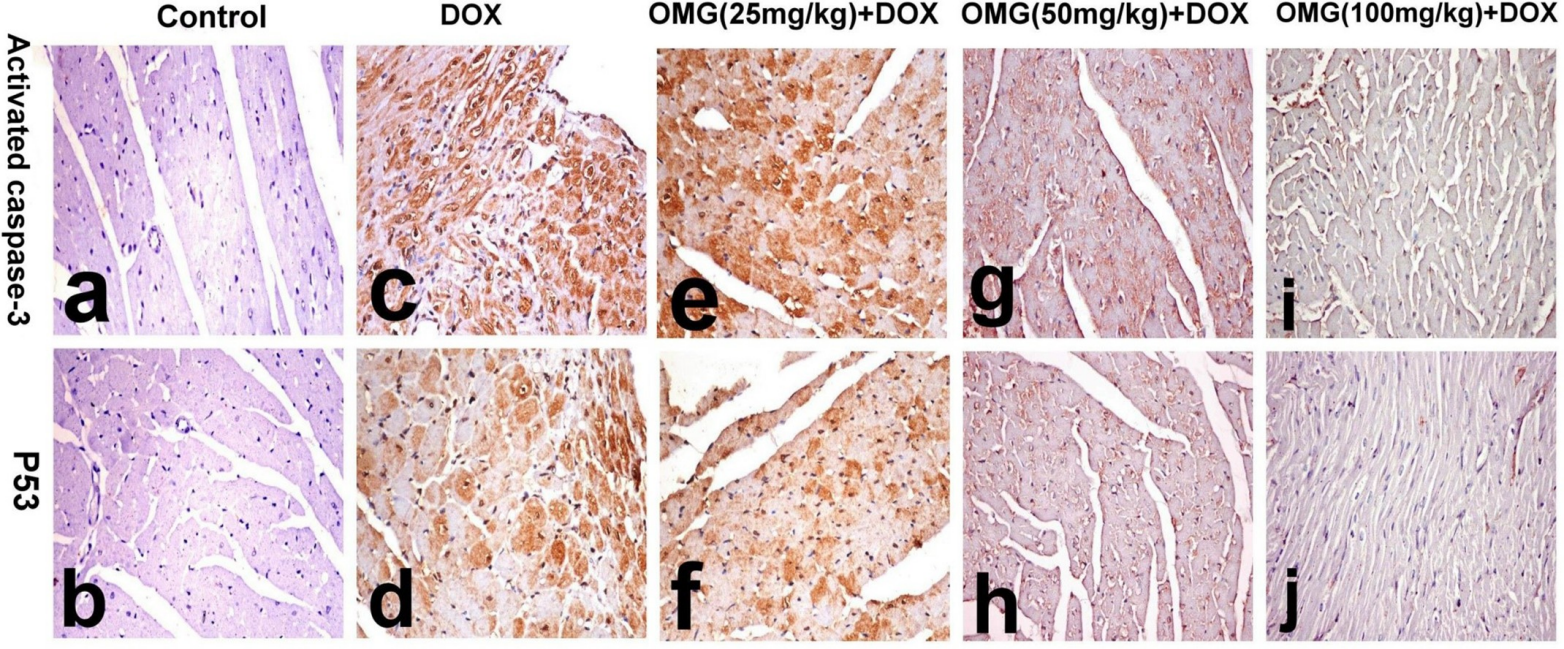
Immunohistochemical staining of activated caspase 3 and P53 in heart tissues of the rats. **(a, b)** normal rats showing no staining, **(c, d)** DOX-treated rats showing diffuse intensely stained cytoplasm and/or nuclei, **(e, f)** OMG (25mg/kg)+DOX showing diffuse positive reaction in the cardiomyocytes, **(g, h)** OMG (50mg/kg)+DOX showing faint immune reaction in the cardiomyocytes, and **(i, j)** OMG (100mg/kg)+DOX showing very faint immune staining of individual cardiomyocytes. (activated caspase 3 immunohistochemical staining for a, c, e, g, and i and P53 immunohistochemical staining for b, d, f, h and j. 40X). DOX, doxorubicin, OMG, omega 3 fatty acids.

**Fig 8:**
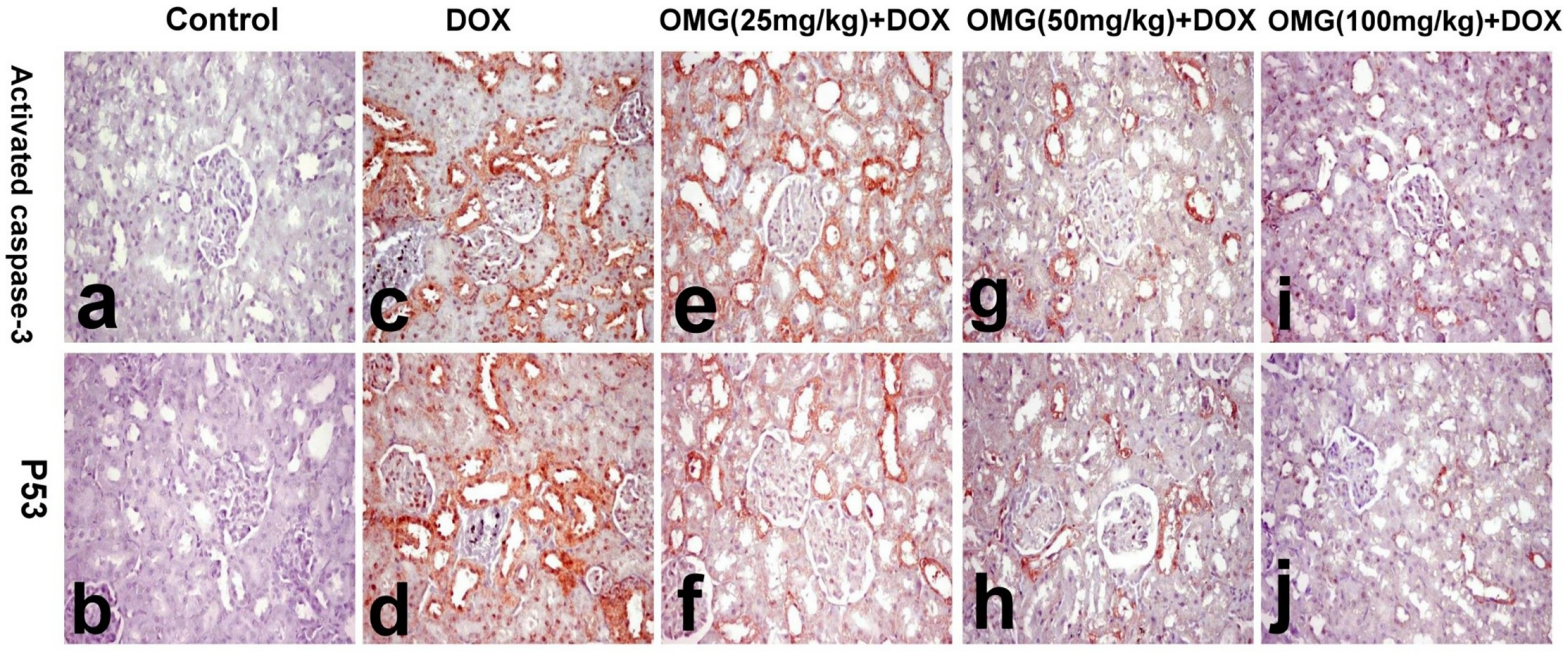
Immunohistochemical staining of activated caspase 3 and P53 in the in heart tissues of the rats. (a, b) normal rats showing no staining, (c, d) DOX-treated rats showing diffuse positive reaction in the glomerular and renal tubular epithelium, (e, f) OMG (25mg/kg) + DOX showing diffuse positive reaction in the renal tubular epithelium,(g, h) OMG (50mg/kg) + DOX showing focal immune staining in the glomerular and renal tubular epithelium, and(i, j) OMG(100mg/kg) + DOX showing faint and focal immune staining. (activated caspase 3 immunohistochemical staining for a, c, e, g, and i and P53 immunohistochemical staining for b, d, f, h and j. 40X). DOX, doxorubicin, OMG, omega 3 fatty acids.

The results of immunohistochemical analysis of activated caspase-3 and P53 demonstrated in the kidneys and heart of the control and treated rats are illustrated in Table 6 and Figs 7 and 8.

**Table 6:** Effect of OMG on cardiac and renal activated caspase 3 and P53 in DOX-treated rats.

Parameters Groups	Activated caspase 3 (% of stained area)		P53 (% of stained area)	
	Heart	kidneys	Heart	kidneys
**Normal**	0.00±0.00	0.00±0.00	0.00±0.00	0.00^a^±0.00
**DOX**	2.00*±0.00	1.60*±0.24	1.60*±0.24	1.60*±0.24
**DOX+OMG (25mg/kg)**	1.40*^@^±0.24	1.40*^@^±0.37	1.20*^@^±0.37	1.24*^@^±0.37
**DOX+OMG (50mg/kg)**	1.00^@^±0.31	1.00^@^±0.37	0.60^@^±0.24	0.80^@^±0.37
**DOX+OMG (100 mg/kg)**	0.60^@^±0.24	0.20^@^±0.20	0.40^@^±0.24	0.40±0.24

Cardiorenal toxicity were induced in rats by a single intraperioneal injection of doxorubicin (DOX; 200 mg/kg,i.p) after 28 consecutive days of oral administration of omega 3 (OMG; 25, 50 and 100 mg/kg) fatty acids. Forty-eight hours after DOX injection, the rats were scarified and the heart and renal were isolated. Tissue specimens from the kidneys and heart of control and treated rats were fixed in 10% neutral buffered formalin and routinely processed. Paraffin embedded sections were dewaxed, rehydrated and incubated in 3% hydrogen peroxide for inhibition of endogenous peroxidase. The sections were then incubated with rabbit anti-cleaved caspase 3 (Abcam, 1:100 Cambridge, MA, USA) and mouse monoclonal anti-P53 (Ventana) as the primary antibodies. Staining was visualized with DAB. Results are the mean ± SEM. Statistical analyses were carried out using ANOVA followed by Tukey's multiple comparisons test.

*, P< 0.05 compared with the normal control

^@^, P< 0.05 compared with the DOX treated rats.

Immunohistochemical staining for activated caspase 3 and P53 revealed no staining in the kidneys and heart of control rats (grade 0). While, marked and diffuse positive reaction for activated caspase 3 and P53 were demonstrated in the glomerular and renal tubular epithelium as well as cardiomyocytes of DOX-treated rats (grade 2). Similarly, intense positive reaction was demonstrated in OMG (25mg/kg) +DOX group (grade 2). On the contrary, the renal tubular epithelium and cardiomyocytes of OMG (50mg/kg) +DOX and OMG (100mg/kg) +DOX groups revealed faint and focal immune staining (grade 1) which is significantly different from DOX- treated group (Figs 7 and 8).

### 3.6. Cytotoxicity assay findings

**Fig 9:**
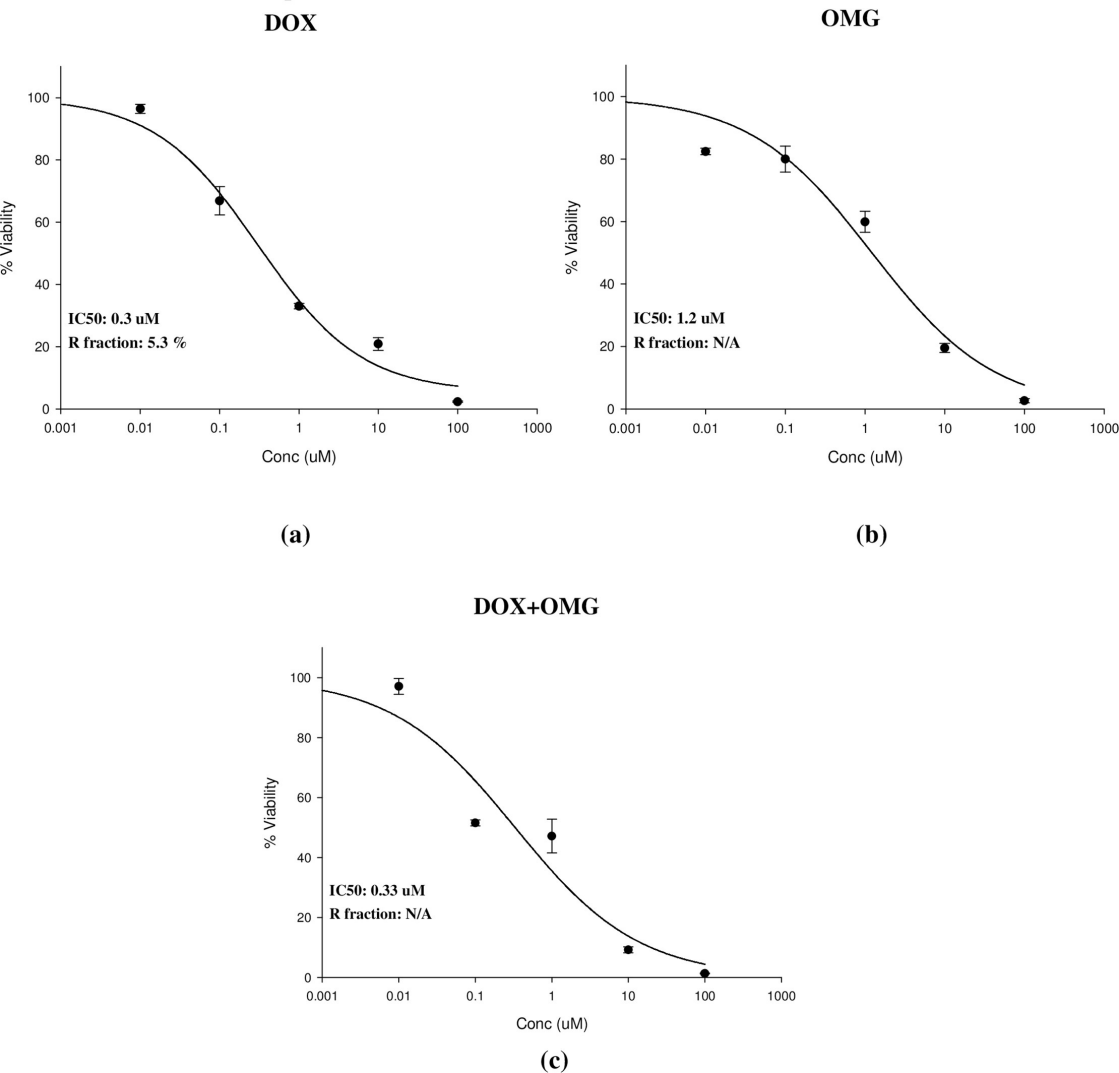
Cytotoxicity of DOX, OMG, and their combination as assessed *in vitro* against human breast cancer MCF7cell line. DOX, doxorubicin, OMG, omega 3 fatty acids; Statistical analysis of IC50 values were calculated from concentration-response curves by Sigma Plot software, version 12.0 (System Software, USA).

DOX exerted gradient cytotoxic activity against MCF7 with increasing concentration; viability started to drop significantly at a concentration of 1 μM. Cellular log kill was gradual in profile with IC50 of 0.3 ± 0.02 μM. Similarly, OMG single treatment exerted gradual cytotoxic activity with increasing concentration; viability started to drop significantly at concentration of 10 μM. OMG has steep cellular log kill profile with IC50 of 1.2 ± 0.02 μM. Equimolar combination of OMG with DOX significantly improved the cytotoxic profile of OMG. IC50 of OMG after combination with DOX was significantly decreased from 1.2 ± 0.02 μ M to 0.33 ± 0.01μ M. However, this combination showed no effect on the cytotoxic profile of DOX (Fig 9).

## 4. Discussion

In this study, single intraperitoneal injection of DOX (200 mg/kg) induced toxic effects on the heart and kidneys of rats. The cardiotoxicity was reflected by the recorded ECG in DOX-treated rats on both conductivity and rhythmicity of cardiac muscle. Both QTc and R-R intervals were elongated with a marked bradycardia, in addition to the elevation of ST height and shortening of T- wave amplitude, indicating the delay in both conductivity and rhythmicity of cardiac cells. Theses current findings in the DOX group were in agreement with previous studies [29,30], and they resemble the typical characteristics of acute DOX-induced cardiotoxicity in human [31]. Moreover, induction of cardiotoxicity by DOX was also confirmed by the observed elevation in serum levels of CK and hsCRP as well as cardiac content of ANP and IL-6. It was also established by the observed histopathological alterations of cardiac tissues. CK and ANP are myocardium-specific protein reported as useful biomarkers for heart diseases [32]. Previous studies reported the increased release of CK and ANP from myocardial tissues with DOX treatment [33–35]. IL-6 is an important cardiac cytokine that is upregulated in the acute phase of cardiac injury [36], and mediates adverse myocardial remodeling [37]. Its elevated levels in relation with acute DOX-induced cardiotoxicity has been demonstrated in other studies [38,39]. Also, hsCRP is a strong indicator of cardiac stress that is accompanying increased rates of cardiovascular diseases [40]. Elevation in hsCRP levels has been reported before in association with DOX-induced cardiotoxicity [41].

In the same way, DOX-induced nephrotoxicity was manifested by increasing serum levels of kidney function biomarkers, urea and creatinine, as well as alteration of histopathological features of renal tissues in DOX-treated rats. Similar findings have been recorded before [1,42,43]. Furthermore, upregulation of renal Nox4 was observed in the current study. NADPH-oxidase (Nox) is an important source of ROS that has many isoforms including Nox1, Nox2, Nox3, and Nox4. In the kidney, Nox4 is highly expressed and plays an important role in nephropathologies through ROS-mediated and non-mediated pathways [10,11]. By stimulating several signaling pathways, Nox4 leads to redox processes including diabetic nephropathy, acute kidney injury, obstructive nephropathy, hypertensive nephropathy, carcinoma of the renal cells and other renal diseases [44]. In line with our findings, upregulation of renal Nox4 with DOX was reported before [9,45]. Previous studies suggested that ROS generated by NADPH oxidases can activate Nrf2 [46,47], which is the redox homeostasis modulator [48]. The expression of Nrf2-dependent genes is upregulated by overexpression of Nox4. This Nox4-directed upregulation was completely attenuated in an Nrf2-null inherited background, indicating the involvement of Nrf2 in the molecular pathway [49]. Moreover, Nrf2 regulation has been recently related to the chemosensitivity of cancer cells, thus this approach is a promising therapy for chemoresistant neoplastic cells [50]. Ryoo et al. [51] has also revealed the involvement of Nrf2 signaling in DOX resistance of cancer stem cell.

Also, increased levels of renal renin was demonstrated in the present study. According to literatures, elevated levels of renal renin are best attributed to reduced renal perfusion and impair glomerular filtration [52]. Decreased renal perfusion releases large quantities of renin as a provoking factor to induce compensatory renal vasoconstriction. This action occur entirely intrarenally, and the vasoconstriction appears to be limited to this vascular bed. Therefore, evaluation of renal the renin, rather than peripherally, gives more accurate indication of renal problem [53]. The current increase in renal renin levels assumes that a part of DOX-induced nephrotoxicity might be secondary to a preceding cardiotoxicity that results in decreasing the cardiac output, as evidenced by ECG in the current study, and consequently the renal perfusion. Supporting this hypothesis, previous data suggested that DOX-induced cardiotoxicity might lead to modulation of blood supply to the kidney and alter xenobiotic detoxification processes, respectively, thus indirectly contributing to DOX-induced nephropathy [8].

On the other hand, our study evidenced that DOX nephrotoxic effect is also a part of a multiorgan damage mediated by DOX mainly through free radical formation. This was demonstrated by deterioration of oxidative stress biomarkers as indicated by a reduction of GSH content and elevation of lipid peroxidation end product, MDA, in both cardiac and renal tissues of DOX-treated rats. These results that are concordant with those from other studies [54,55]. Exertion of DOX-induced oxidative stress occurs as a result of DOX reduction by the mitochondrial enzymes following its cellular uptake; a reaction that results in generation of abundant ROS [33,56]. This may explain that DOX-induced toxicity mostly affects cells with a huge number of mitochondria, including cardiac and renal cells [30]. Furthermore, DOX also decreases the levels of endogenous antioxidant, such as GSH. Therefore, the low concentrations of such proteins in heart and kidney tissues gives another explanation of the high susceptibility of both organs to DOX toxicity [57].

Moreover, the current immunohistochemical findings revealed increased expression of apoptotic markers such as caspase-3 and P53 in the heart and kidneys of DOX-treated rats. Accumulating evidence supports induction of apoptosis as a key mechanism for DOX-induced cardiorenal toxicity [58,59]. DOX-induced elevated levels of free radicles cause intercalation into DNA leading to apoptosis [60–64]. These findings was empathized by Mizutani *et al* who exhibited activation of caspase-3 in human promyelocytic leukemia (HL60) cells treated with DOX resulting in cell death [65]. Interestingly, in the study of Mizutani *et al*, apoptosis was identified to be mediating through different mechanisms including Nox activation. This explains our finding where renal apoptosis was accompanied by elevated expression of Nox4, suggesting the latter as one of the driving mechanism leading to DOX-induced apoptosis.

However, pretreatment of DOX-injected rats with with OMG (25, 50 or 100 mg/kg; p.o.) showed a marked ameliorative effect of the acute cardiorenal toxicity in a dose-dependent manner. In agreement, the protective effect of OMG against DOX-induced cardiac and renal toxicities has been described in other studies [66,67]. The data of the current study revealed an improvement in ECG, cardiac and renal function biomarkers and histopathological findings by OMG in DOX-treated rats. Several mechanisms of cardio- and reno- protective effects of OMG against different heart and kidney diseases have been reported, including their advantage on platelet function, cytokine production, fibrinolysis, and inflammatory factors [68–71]. Moreover, the present study confirmed the established anti-oxidant and anti-apoptotic activity of OMG [72–74] by showing an improvement in oxidative and apoptotic biomarkers in both heart and kidney tissues of DOX-injected rats when pretreated with OMG. These findings indicated those activities as protective mechanisms of OMG. Moreover, a decline of Nox4 expression was observed with OMG pretreatment. Interestingly, this points out a role of Nox4 inhibition in the protective effect of OMG against DOX-induced renal toxicity, especially, when considered the direct effect of OMG on Nox in different organs that has been described [75,76]. Moreover, this finding indicates a protective role of OMG against Nox4-Nrf2 redox imbalance, suggesting it as a potential agent to be used in conjunction with the chemotherapeutic agent, DOX, to reduce the probability of chemoresistance. It has been previously found that OMG chemosensitize multidrug resistant colon cancer cells [77]. Further investigation is still required to assess this effect of OMG and this would be of potential importance because pharmacological inhibition of Nox4 has received massive consideration as a therapeutic strategy for many kidney diseases [78].

An important point in using a protective agent against DOX-induced deleterious effects is to confirm that administration of this protective does not interfere with DOX antitumor activity [79]. In this study, preservation of the cytotoxic activity of DOX, when tested on MCF7 cell line in the presence of OMG, was observed. In line with our findings, data reveals that OMG do not only have any negative effect on the antitumor activity of conventional cancer therapies, however, they can also increase the sensitivity of tumor cells to these drugs and improve their efficacy, especially against cancers resistant to treatment [80]. Stimulatingly, a cytotoxic activity of OMG alone has been observed in the current in-vitro study. This result is in agreement with the studies reported a selective cytotoxic activity of OMG towards cancer cells with little or no toxicity on normal cells [81,82].

## 5. Conclusion

In conclusion, the findings of the current study revealed induction of acute cardiorenal tocicity in rat by a single intraperitoneal injection of DOX (200 mg/kg) that is characterized by oxidative stress and induction of apoptosis in both heart and kidney tissues. Activation of Nox4 that observed in the renal tissues suggested its role in DOX oxidative and proapoptoyic effects. Elevation of renal renin levels that was accompanied by defective cardiac functions hypothesized that part of DOX-induced renal toxicity occurs secondary to impaired renal perfusion due to DOX-cardiotoxic outcome. The study also showed that pretreatment with OMG protected the rats against DOX-induced cardiorenal toxicity. It demonstrated the anti-oxidant and anti-apoptotic activities of OMG as underlying mechanisms for this reported protective effect, and proposed the involvement of Nox4 inhibition. The study confirmed the preservation of DOX cytotoxic activity *in vitro* when used with OMG. Therefore, this study suggests OMG as a protective agent against acute DOX‐induced cardiorenal damage, with no negative influence on the latter required antitumor activity.

## References

[1] Nagai K, Fukuno S, Otani K, Nagamine Y, Omotani S, Hatsuda Y, et al Prevention of Doxorubicin-Induced Renal Toxicity by Theanine in Rats. Pharmacology 2018; 101: 219–24. 10.1159/000486625 29393264

[2] Injac R, Strukelj B. Recent advances in protection against doxorubicin-induced toxicity. Technol Cancer Res Treat 2008; 7: 497–516. 10.1177/153303460800700611 19044329

[3] Kitada M, Horie T, Awazu S. Chemiluminescence associated with doxorubicin-induced lipid peroxidation in rat heart mitochondria. Biochem Pharmacol 1994; 48: 93–9. 8043035

[4] Saleem MT, Chetty MC, Kavimani S. Antioxidants and tumor necrosis factor alpha-inhibiting activity of sesame oil against doxorubicin-induced cardiotoxicity. Ther Adv Cardiovasc Dis 2014; 8: 4–11. 10.1177/1753944713516532 24441175

[5] Patel N, Joseph C, Corcoran GB, Ray SD. Silymarin modulates doxorubicin-induced oxidative stress, Bcl-xL and p53 expression while preventing apoptotic and necrotic cell death in the liver. Toxicol Appl Pharmacol 2010; 245: 143–52. 10.1016/j.taap.2010.02.002 20144634

[6] Thorn CF, Oshiro C, Marsh S, Hernandez-Boussard T, McLeod H, Klein TE, et al Doxorubicin pathways: pharmacodynamics and adverse effects. Pharmacogenet Genomics 2011; 21: 440–6. 10.1097/FPC.0b013e32833ffb56 21048526 PMC3116111

[7] Giri SN, Al-Bayati MA, Du X, Schelegle E, Mohr FC, Margolin SB. Amelioration of doxorubicin-induced cardiac and renal toxicity by pirfenidone in rats. Cancer Chemother Pharmacol 2004; 53: 141–50. 10.1007/s00280-003-0703-z 14564477

[8] Injac R, Boskovic M, Perse M, Koprivec-Furlan E, Cerar A, Djordjevic A, et al Acute doxorubicin nephrotoxicity in rats with malignant neoplasm can be successfully treated with fullerenol C60(OH)24 via suppression of oxidative stress. Pharmacol Rep 2008; 60: 742–9. 19066422

[9] Elsherbiny NM, El-Sherbiny M. Thymoquinone attenuates Doxorubicin-induced nephrotoxicity in rats: Role of Nrf2 and NOX4. Chem Biol Interact 2014; 223: 102–8. 10.1016/j.cbi.2014.09.015 25268985

[10] Sedeek M, Nasrallah R, Touyz RM, Hebert RL. NADPH oxidases, reactive oxygen species, and the kidney: friend and foe. J Am Soc Nephrol 2013; 24: 1512–8. 10.1681/ASN.2012111112 23970124 PMC3785272

[11] Gill PS, Wilcox CS. NADPH oxidases in the kidney. Antioxid Redox Signal 2006; 8: 1597–607. 10.1089/ars.2006.8.1597 16987014

[12] Freitas RDS, Campos MM. Protective Effects of Omega-3 Fatty Acids in Cancer-Related Complications. Nutrients 2019; 11 10.3390/nu11050945 31035457 PMC6566772

[13] de Batlle J, Sauleda J, Balcells E, Gomez FP, Mendez M, Rodriguez E, et al Association between Omega3 and Omega6 fatty acid intakes and serum inflammatory markers in COPD. J Nutr Biochem 2012; 23: 817–21. 10.1016/j.jnutbio.2011.04.005 21889886

[14] Avramovic N, Dragutinovic V, Krstic D, Colovic M, Trbovic A, de Luka S, et al The effects of omega 3 fatty acid supplementation on brain tissue oxidative status in aged wistar rats. Hippokratia 2012; 16: 241–5. 23935291 PMC3738731

[15] Swanson D, Block R, Mousa SA. Omega-3 fatty acids EPA and DHA: health benefits throughout life. Adv Nutr 2012; 3: 1–7. 10.3945/an.111.000893 22332096 PMC3262608

[16] Ebrahimi M, Ghayour-Mobarhan M, Rezaiean S, Hoseini M, Parizade SM, Farhoudi F, et al Omega-3 fatty acid supplements improve the cardiovascular risk profile of subjects with metabolic syndrome, including markers of inflammation and auto-immunity. Acta Cardiol 2009; 64: 321–7. 10.2143/AC.64.3.2038016 19593941

[17] Khan S, Minihane AM, Talmud PJ, Wright JW, Murphy MC, Williams CM, et al Dietary long-chain n-3 PUFAs increase LPL gene expression in adipose tissue of subjects with an atherogenic lipoprotein phenotype. J Lipid Res 2002; 43: 979–85. 12032174

[18] Oscarsson J, Hurt-Camejo E. Omega-3 fatty acids eicosapentaenoic acid and docosahexaenoic acid and their mechanisms of action on apolipoprotein B-containing lipoproteins in humans: a review. Lipids Health Dis 2017; 16: 149 10.1186/s12944-017-0541-3 28797250 PMC5553798

[19] El-Ashmawy NE, Khedr NF, El-Bahrawy HA, Helal SA. Upregulation of PPAR-gamma mediates the renoprotective effect of omega-3 PUFA and ferulic acid in gentamicin-intoxicated rats. Biomed Pharmacother 2018; 99: 504–10. 10.1016/j.biopha.2018.01.036 29665653

[20] Hassan IR, Gronert K. Acute changes in dietary omega-3 and omega-6 polyunsaturated fatty acids have a pronounced impact on survival following ischemic renal injury and formation of renoprotective docosahexaenoic acid-derived protectin D1. J Immunol 2009; 182: 3223–32. 10.4049/jimmunol.0802064 19234220

[21] Hajrasouliha AR, Tavakoli S, Jabehdar-Maralani P, Shafaroodi H, Borhani AA, Houshmand G, et al Resistance of cholestatic rats against epinephrine-induced arrhythmia: the role of nitric oxide and endogenous opioids. Eur J Pharmacol 2004; 499: 307–13. 10.1016/j.ejphar.2004.07.099 15381053

[22] Jensen RA, Acton EM, Peters JH. Doxorubicin cardiotoxicity in the rat: comparison of electrocardiogram, transmembrane potential, and structural effects. J Cardiovasc Pharmacol 1984; 6: 186–200. 6199603

[23] Wellington D, Mikaelian I, Singer L. Comparison of ketamine-xylazine and ketamine-dexmedetomidine anesthesia and intraperitoneal tolerance in rats. J Am Assoc Lab Anim Sci 2013; 52: 481–7. 23849447 PMC3725934

[24] Szasz G, Borner U, Busch EW, Bablok W. [Enzymatic assay of creatinine in serum: comparison with Jaffe methods (author's transl)]. J Clin Chem Clin Biochem 1979; 17: 683–7. 547029

[25] Fawcett JK, Scott JE. A rapid and precise method for the determination of urea. J Clin Pathol 1960; 13: 156–9. 10.1136/jcp.13.2.156 13821779 PMC480024

[26] Bartles JR, Pardos BT, Frazier WA. Reconstitution of discoidin hemagglutination activity by lipid extracts of Dictyostelium discoideum cells. J Biol Chem 1979; 254: 3156–9. 429337

[27] Reagan WJ, York M, Berridge B, Schultze E, Walker D, Pettit S. Comparison of cardiac troponin I and T, including the evaluation of an ultrasensitive assay, as indicators of doxorubicin-induced cardiotoxicity. Toxicol Pathol 2013; 41: 1146–58. 10.1177/0192623313482056 23531791

[28] Panjrath GS, Patel V, Valdiviezo CI, Narula N, Narula J, Jain D. Potentiation of Doxorubicin cardiotoxicity by iron loading in a rodent model. J Am Coll Cardiol 2007; 49: 2457–64. 10.1016/j.jacc.2007.02.060 17599610

[29] Goyal V, Bews H, Cheung D, Premecz S, Mandal S, Shaikh B, et al The Cardioprotective Role of N-Acetyl Cysteine Amide in the Prevention of Doxorubicin and Trastuzumab-Mediated Cardiac Dysfunction. Can J Cardiol 2016; 32: 1513–9. 10.1016/j.cjca.2016.06.002 27650929

[30] Mustafa HN, Hegazy GA, Awdan SAE, AbdelBaset M. Protective role of CoQ10 or L-carnitine on the integrity of the myocardium in doxorubicin induced toxicity. Tissue Cell 2017; 49: 410–26. 10.1016/j.tice.2017.03.007 28410798

[31] Licata S, Saponiero A, Mordente A, Minotti G. Doxorubicin metabolism and toxicity in human myocardium: role of cytoplasmic deglycosidation and carbonyl reduction. Chem Res Toxicol 2000; 13: 414–20. 10.1021/tx000013q 10813659

[32] Kim K, Chini N, Fairchild DG, Engle SK, Reagan WJ, Summers SD, et al Evaluation of Cardiac Toxicity Biomarkers in Rats from Different Laboratories. Toxicol Pathol 2016; 44: 1072–83. 10.1177/0192623316668276 27638646 PMC5330931

[33] Ruan Y, Dong C, Patel J, Duan C, Wang X, Wu X, et al SIRT1 suppresses doxorubicin-induced cardiotoxicity by regulating the oxidative stress and p38MAPK pathways. Cell Physiol Biochem 2015; 35: 1116–24. 10.1159/000373937 25766524

[34] Zhang YW, Shi J, Li YJ, Wei L. Cardiomyocyte death in doxorubicin-induced cardiotoxicity. Arch Immunol Ther Exp (Warsz) 2009; 57: 435–45. 19866340 10.1007/s00005-009-0051-8 PMC2809808

[35] Wenningmann N, Knapp M, Ande A, Vaidya TR, Ait-Oudhia S. Insights into Doxorubicin-induced Cardiotoxicity: Molecular Mechanisms, Preventive Strategies, and Early Monitoring. Mol Pharmacol 2019; 96: 219–32. 10.1124/mol.119.115725 31164387

[36] Deten A, Volz HC, Briest W, Zimmer HG. Cardiac cytokine expression is upregulated in the acute phase after myocardial infarction. Experimental studies in rats. Cardiovasc Res 2002; 55: 329–40. 10.1016/s0008-6363(02)00413-3 12123772

[37] Melendez GC, McLarty JL, Levick SP, Du Y, Janicki JS, Brower GL. Interleukin 6 mediates myocardial fibrosis, concentric hypertrophy, and diastolic dysfunction in rats. Hypertension 2010; 56: 225–31. 10.1161/HYPERTENSIONAHA.109.148635 20606113 PMC2921860

[38] Hijazi MA, Jambi HA, Aljehany BM, Althaiban MA. Potential Protective Effect of Achillea fragrantissima against Adriamycin-Induced Cardiotoxicity in Rats via an Antioxidant and Anti-Inflammatory Pathway. Biomed Res Int 2019; 2019: 5269074 10.1155/2019/5269074 31317032 PMC6601502

[39] Sun Z, Yan B, Yu WY, Yao X, Ma X, Sheng G, et al Vitexin attenuates acute doxorubicin cardiotoxicity in rats via the suppression of oxidative stress, inflammation and apoptosis and the activation of FOXO3a. Exp Ther Med 2016; 12: 1879–84. 10.3892/etm.2016.3518 27588105 PMC4997971

[40] Singh SK, Suresh MV, Voleti B, Agrawal A. The connection between C-reactive protein and atherosclerosis. Ann Med 2008; 40: 110–20. 10.1080/07853890701749225 18293141 PMC3364506

[41] Fadillioglu E, Oztas E, Erdogan H, Yagmurca M, Sogut S, Ucar M, et al Protective effects of caffeic acid phenethyl ester on doxorubicin-induced cardiotoxicity in rats. J Appl Toxicol 2004; 24: 47–52. 10.1002/jat.945 14745846

[42] Sindhu ER, Nithya TR, Binitha PP, Kuttan R. Amelioration of Doxorubicin-Induced Cardiac and Renal Toxicity by Oxycarotenoid Lutein and Its Mechanism of Action. J Environ Pathol Toxicol Oncol 2016; 35: 237–47. 10.1615/JEnvironPatholToxicolOncol.2016014010 27910779

[43] Szalay CI, Erdelyi K, Kokeny G, Lajtar E, Godo M, Revesz C, et al Oxidative/Nitrative Stress and Inflammation Drive Progression of Doxorubicin-Induced Renal Fibrosis in Rats as Revealed by Comparing a Normal and a Fibrosis-Resistant Rat Strain. PLoS One 2015; 10: e0127090 10.1371/journal.pone.0127090 26086199 PMC4473269

[44] Yang Q, Wu FR, Wang JN, Gao L, Jiang L, Li HD, et al Nox4 in renal diseases: An update. Free Radic Biol Med 2018; 124: 466–72. 10.1016/j.freeradbiomed.2018.06.042 29969717

[45] Guo J, Ananthakrishnan R, Qu W, Lu Y, Reiniger N, Zeng S, et al RAGE mediates podocyte injury in adriamycin-induced glomerulosclerosis. J Am Soc Nephrol 2008; 19: 961–72. 10.1681/ASN.2007101109 18256352 PMC2386730

[46] Churchman AT, Anwar AA, Li FY, Sato H, Ishii T, Mann GE, et al Transforming growth factor-beta1 elicits Nrf2-mediated antioxidant responses in aortic smooth muscle cells. J Cell Mol Med 2009; 13: 2282–92. 10.1111/j.1582-4934.2009.00874.x 19674192 PMC6529974

[47] Lee IT, Wang SW, Lee CW, Chang CC, Lin CC, Luo SF, et al Lipoteichoic acid induces HO-1 expression via the TLR2/MyD88/c-Src/NADPH oxidase pathway and Nrf2 in human tracheal smooth muscle cells. J Immunol 2008; 181: 5098–110. 10.4049/jimmunol.181.7.5098 18802114

[48] Saleh DO, Mansour DF, Hashad IM, Bakeer RM. Effects of sulforaphane on D-galactose-induced liver aging in rats: Role of keap-1/nrf-2 pathway. Eur J Pharmacol 2019; 855: 40–9. 10.1016/j.ejphar.2019.04.043 31039346

[49] Brewer AC, Murray TV, Arno M, Zhang M, Anilkumar NP, Mann GE, et al Nox4 regulates Nrf2 and glutathione redox in cardiomyocytes in vivo. Free Radic Biol Med 2011; 51: 205–15. 10.1016/j.freeradbiomed.2011.04.022 21554947 PMC3112490

[50] Mukhopadhyay S, Goswami D, Adiseshaiah PP, Burgan W, Yi M, Guerin TM, et al Undermining Glutaminolysis Bolsters Chemotherapy While NRF2 Promotes Chemoresistance in KRAS-Driven Pancreatic Cancers. Cancer Res 2020; 80: 1630–43. 10.1158/0008-5472.CAN-19-1363 31911550 PMC7185043

[51] Ryoo IG, Kim G, Choi BH, Lee SH, Kwak MK. Involvement of NRF2 Signaling in Doxorubicin Resistance of Cancer Stem Cell-Enriched Colonospheres. Biomol Ther (Seoul) 2016; 24: 482–8. 10.4062/biomolther.2016.145 27582554 PMC5012872

[52] Tang J, Wysocki J, Ye M, Valles PG, Rein J, Shirazi M, et al Urinary Renin in Patients and Mice With Diabetic Kidney Disease. Hypertension 2019; 74: 83–94. 10.1161/HYPERTENSIONAHA.119.12873 31079532 PMC6561816

[53] Smith IK. The role of renin in renal failure associated with hepatic failure. Postgrad Med J 1975; 51: 506–8. 10.1136/pgmj.51.598.506 1234331 PMC2496270

[54] Cappetta D, De Angelis A, Sapio L, Prezioso L, Illiano M, Quaini F, et al Oxidative Stress and Cellular Response to Doxorubicin: A Common Factor in the Complex Milieu of Anthracycline Cardiotoxicity. Oxid Med Cell Longev 2017; 2017: 1521020 10.1155/2017/1521020 29181122 PMC5664340

[55] Zhang QL, Yang JJ, Zhang HS. Carvedilol (CAR) combined with carnosic acid (CAA) attenuates doxorubicin-induced cardiotoxicity by suppressing excessive oxidative stress, inflammation, apoptosis and autophagy. Biomed Pharmacother 2019; 109: 71–83. 10.1016/j.biopha.2018.07.037 30396094

[56] Carvalho PB, Goncalves AF, Alegre PH, Azevedo PS, Roscani MG, Bergamasco CM, et al Pamidronate Attenuates Oxidative Stress and Energetic Metabolism Changes but Worsens Functional Outcomes in Acute Doxorubicin-Induced Cardiotoxicity in Rats. Cell Physiol Biochem 2016; 40: 431–42. 10.1159/000452558 27889760

[57] Roca-Alonso L, Pellegrino L, Castellano L, Stebbing J. Breast cancer treatment and adverse cardiac events: what are the molecular mechanisms? Cardiology 2012; 122: 253–9. 10.1159/000339858 22907032

[58] Drimal J, Zurova-Nedelcevova J, Knezl V, Sotnikova R, Navarova J. Cardiovascular toxicity of the first line cancer chemotherapeutic agents: doxorubicin, cyclophosphamide, streptozotocin and bevacizumab. Neuro Endocrinol Lett 2006; 27 Suppl 2: 176–9. 17159809

[59] Renu K V GA, P BT, Arunachalam S. Molecular mechanism of doxorubicin-induced cardiomyopathy—An update. Eur J Pharmacol 2018; 818: 241–53. 10.1016/j.ejphar.2017.10.043 29074412

[60] Takemura G, Fujiwara H. Doxorubicin-induced cardiomyopathy from the cardiotoxic mechanisms to management. Prog Cardiovasc Dis 2007; 49: 330–52. 10.1016/j.pcad.2006.10.002 17329180

[61] Gewirtz DA. A critical evaluation of the mechanisms of action proposed for the antitumor effects of the anthracycline antibiotics adriamycin and daunorubicin. Biochem Pharmacol 1999; 57: 727–41. 10.1016/s0006-2952(98)00307-4 10075079

[62] Kalyanaraman B, Perez-Reyes E, Mason RP. Spin-trapping and direct electron spin resonance investigations of the redox metabolism of quinone anticancer drugs. Biochim Biophys Acta 1980; 630: 119–30. 10.1016/0304-4165(80)90142-7 6248123

[63] Doroshow JH. Effect of anthracycline antibiotics on oxygen radical formation in rat heart. Cancer Res 1983; 43: 460–72. 6293697

[64] Bristow MR, Sageman WS, Scott RH, Billingham ME, Bowden RE, Kernoff RS, et al Acute and chronic cardiovascular effects of doxorubicin in the dog: the cardiovascular pharmacology of drug-induced histamine release. J Cardiovasc Pharmacol 1980; 2: 487–515. 10.1097/00005344-198009000-00002 6157945

[65] Mizutani H, Tada-Oikawa S, Hiraku Y, Kojima M, Kawanishi S. Mechanism of apoptosis induced by doxorubicin through the generation of hydrogen peroxide. Life Sci 2005; 76: 1439–53. 10.1016/j.lfs.2004.05.040 15680309

[66] Uygur R, Aktas C, Tulubas F, Alpsoy S, Topcu B, Ozen OA. Cardioprotective effects of fish omega-3 fatty acids on doxorubicin-induced cardiotoxicity in rats. Hum Exp Toxicol 2014; 33: 435–45. 10.1177/0960327113493304 24064909

[67] Tulubas F, Gurel A, Oran M, Topcu B, Caglar V, Uygur E. The protective effects of omega-3 fatty acids on doxorubicin-induced hepatotoxicity and nephrotoxicity in rats. Toxicol Ind Health 2015; 31: 638–44. 10.1177/0748233713483203 23512535

[68] Desnoyers M, Gilbert K, Rousseau G. Cardioprotective Effects of Omega-3 Polyunsaturated Fatty Acids: Dichotomy between Experimental and Clinical Studies. Mar Drugs 2018; 16 10.3390/md16070234 29996474 PMC6071068

[69] Adkins Y, Kelley DS. Mechanisms underlying the cardioprotective effects of omega-3 polyunsaturated fatty acids. J Nutr Biochem 2010; 21: 781–92. 10.1016/j.jnutbio.2009.12.004 20382009

[70] Dimitrow PP, Jawien M. Pleiotropic, cardioprotective effects of omega-3 polyunsaturated fatty acids. Mini Rev Med Chem 2009; 9: 1030–9. 10.2174/138955709788922638 19689400

[71] Brown SA, Brown CA, Crowell WA, Barsanti JA, Allen T, Cowell C, et al Beneficial effects of chronic administration of dietary omega-3 polyunsaturated fatty acids in dogs with renal insufficiency. J Lab Clin Med 1998; 131: 447–55. 10.1016/s0022-2143(98)90146-9 9605110

[72] Sinha RA, Khare P, Rai A, Maurya SK, Pathak A, Mohan V, et al Anti-apoptotic role of omega-3-fatty acids in developing brain: perinatal hypothyroid rat cerebellum as apoptotic model. Int J Dev Neurosci 2009; 27: 377–83. 10.1016/j.ijdevneu.2009.02.003 19460632

[73] Xu F, Song Y, Guo A. Anti-Apoptotic Effects of Docosahexaenoic Acid in IL-1beta-Induced Human Chondrosarcoma Cell Death through Involvement of the MAPK Signaling Pathway. Cytogenet Genome Res 2019; 158: 17–24. 10.1159/000500290 31261155

[74] Hajianfar H, Paknahad Z, Bahonar A. The effect of omega-3 supplements on antioxidant capacity in patients with type 2 diabetes. Int J Prev Med 2013; 4: S234–8. 23776730 PMC3678224

[75] Shen J, Rastogi R, Guan L, Li F, Du H, Geng X, et al Omega-3 fatty acid supplement reduces activation of NADPH oxidase in intracranial atherosclerosis stenosis. Neurol Res 2018; 40: 499–507. 10.1080/01616412.2018.1451290 29576013

[76] Niazi ZR, Silva GC, Ribeiro TP, Leon-Gonzalez AJ, Kassem M, Mirajkar A, et al EPA:DHA 6:1 prevents angiotensin II-induced hypertension and endothelial dysfunction in rats: role of NADPH oxidase- and COX-derived oxidative stress. Hypertens Res 2017; 40: 966–75. 10.1038/hr.2017.72 28878301

[77] Gelsomino G, Corsetto PA, Campia I, Montorfano G, Kopecka J, Castella B, et al Omega 3 fatty acids chemosensitize multidrug resistant colon cancer cells by down-regulating cholesterol synthesis and altering detergent resistant membranes composition. Mol Cancer 2013; 12: 137 10.1186/1476-4598-12-137 24225025 PMC4225767

[78] Rajaram RD, Dissard R, Jaquet V, de Seigneux S. Potential benefits and harms of NADPH oxidase type 4 in the kidneys and cardiovascular system. Nephrol Dial Transplant 2019; 34: 567–76. 10.1093/ndt/gfy161 29931336

[79] Jagetia GC, Reddy TK, Malagi KJ, Nayak BS, Naidu MB, Ravikiran PB, et al Antarth, a polyherbal preparation protects against the doxorubicin-induced toxicity without compromising its Antineoplastic activity. Phytother Res 2005; 19: 772–8. 10.1002/ptr.1713 16220569

[80] D'Eliseo D, Velotti F. Omega-3 Fatty Acids and Cancer Cell Cytotoxicity: Implications for Multi-Targeted Cancer Therapy. J Clin Med 2016; 5 10.3390/jcm5020015 26821053 PMC4773771

[81] Laviano A, Rianda S, Molfino A, Rossi Fanelli F. Omega-3 fatty acids in cancer. Curr Opin Clin Nutr Metab Care 2013; 16: 156–61. 10.1097/MCO.0b013e32835d2d99 23299701

[82] Gu Z, Shan K, Chen H, Chen YQ. n-3 Polyunsaturated Fatty Acids and their Role in Cancer Chemoprevention. Curr Pharmacol Rep 2015; 1: 283–94. 10.1007/s40495-015-0043-9 26457243 PMC4596534

